# Air pressure as a driver of plant-specific microbial responses in the rhizosphere

**DOI:** 10.1186/s40793-025-00805-3

**Published:** 2025-11-18

**Authors:** Theresa Rzehak, Nadine Praeg, Andreas Meul, Silvia Lembo, Bouchra El Omari, Matteo Dainese, Georg Niedrist, Paul Illmer

**Affiliations:** 1 Department of Microbiology, Universität Innsbruck, Innsbruck, Austria; 2 Institute for Alpine Environment, Eurac Research, Bolzano, Bozen, Italy; 3 Department of Biotechnology, University of Verona, Verona, Italy

## Abstract

In response to climate change, plants in mountain regions are shifting their distribution ranges upward, exposing them to novel abiotic conditions such as reduced atmospheric pressure. While these changes are likely to affect plant physiology, their impact on plant-associated microorganisms in the rhizosphere has not yet been investigated. In this study, we used the terraXcube Ecotron facility to experimentally discriminate air pressure from other elevation-related factors like humidity and temperature, and to assess its influence on the rhizosphere microbiota of three plant species: a grass (*Brachipodium rupestre*), a forb (*Hieracium pilosella*), and a legume (*Trifolium pratense*). Plants were grown under controlled environmental conditions at four simulated elevations (260, 1500, 2500, and 4000 m a.s.l.), corresponding to pressure levels of 98, 85, 75, and 62 kPa, respectively. Microbial biomass and activity were significantly influenced by air pressure, but in a plant-specific manner. In addition, air pressure also led to notable and plant-specific shifts in the community composition of prokaryotes and, to a lesser extent, fungi. Redundancy analysis identified air pressure as a central predictor of these rhizosphere community shifts. Notably, no correlations were detected between microbial community composition and morphological and physiological plant traits, suggesting that air pressure should directly affect microorganisms, independently of plant-mediated effects. This study demonstrates that even under constant temperature and humidity, air pressure alone can restructure rhizosphere microbial communities, highlighting a critical yet often overlooked driver of plant–microbe dynamics during uphill range shifts. Whether such alterations in the rhizosphere microbiota ultimately enhance or impair soil chemistry, plant health, and ecosystem functioning remains an important question for future research.

**Supplementary Information:**

The online version contains supplementary material available at 10.1186/s40793-025-00805-3.

## Introduction

The effects of global warming are most evident in ecosystems of the Alpine region, where more rapid changes in temperature occur than at lower elevations, and glacier loss, changes in snow cover extent and duration, and increased natural hazards underscore the severity of climate change [1–3]. In response to rising temperatures, species may adapt their life cycles, face local or global extinction, or shift their ranges upwards to higher elevations to follow their thermal niches [4–7]. In the European Alps, several plant species have already migrated from areas they previously inhabited to higher elevations in response to changing thermal conditions [8]. Consequently, a notable change in vegetation composition is anticipated [8].

However, plant health and establishment in new habitats are tightly coupled to plant-associated microorganisms [9, 10]. Especially under stress conditions, plants depend on their microbiota and can even recruit a stress-resistance-promoting microbiome to the rhizosphere [11, 12]. Within the rhizosphere—the soil compartment surrounding plant roots that is directly influenced by the plant—microorganisms live in close interaction with their host plants. Soil microorganisms preferentially colonize this area, as plants deposit nutritious root exudates (e.g. complex organic compounds, but also amino acids and sugars) [9]. In exchange for these nutrients, rhizosphere microorganisms support plant growth, e.g. under biotic stress, by influencing the plant immune system [9], by regulating plant growth [13], by impacting plant flowering time [14], and by shaping plant community dynamics [15]. Therefore, microorganisms play a central role in plant adaptation to environmental conditions and stressors [16] and impact the establishment of upwards migrated plants at higher elevational levels [17]. This is particularly important because, regardless of dispersal ability or migration rate, all species tracking their thermal niches to higher elevations encounter novel environmental conditions. Upward-migrating species face lower temperatures, increased radiation, and reduced air pressure [18]. In the context of global warming, the role of reduced air pressure has often been overlooked [19, 20]. At sea level, the average atmospheric pressure is about 100 kPa, but decreases by ~ 11% with each kilometer of elevation gain, reaching about 50 kPa over Central Europe at about 5500 m a.s.l. [18, 21]. This decline also lowers the partial pressure of all atmospheric gases [18], thereby affecting key physical parameters such as vapor pressure deficit (VPD), gas diffusivity, and partial pressure of all atmospheric gases. These changes directly influence fundamental processes, including photosynthetic activity, respiration, and evapotranspiration [22]. For example, VPD, a determinant of plant photosynthesis, usually increases with elevation, but it depends on temperature [22, 23]. At the same time, reduced air pressure increases the diffusivity of water vapor in the air, which in turn can lead to higher transpiration in plants [18, 24].

Among the few studies that have assessed the direct effect of air pressure on plants [25–28], microbial responses to low air pressure remain poorly understood [29]. Microbial cells may undergo swelling or shrinkage of cell volume, variations in biomolecule and membrane structure and changes in gas diffusion due to altered pressure [29]. Previous studies identified a critical threshold of 10 kPa, below which bacterial growth rates and cell counts are hampered [30, 31]. Above this pressure threshold, microbial growth appears unaffected, provided that gas availability and water content are not limiting [30, 32]. Nevertheless, data on microbial responses to low atmospheric pressure are scarce, particularly under near-natural conditions [29]. In contrast, numerous studies have investigated the impact of high pressure on microorganisms, suggesting that opposite effects may occur under reduced pressure [30, 33–35].

Most existing studies on microbial responses to air pressure have relied on pure cultures in controlled laboratory settings. Similar to plant physiology experiments, they often tested extremely low pressures, far below those found on Earth and relevant only to outer space environments [26, 29]. But, to better predict air pressure responses of microorganisms and plants in the Alpine region, experimental data along moderate air pressure gradients are necessary. Furthermore, to obtain a comprehensive understanding of Alpine ecosystem functioning under changing climatic conditions, it is essential to test the responses of plants and microorganisms to environmental stressors in combination [36], as microbial communities can mitigate the impact of environmental stressors on plants [37, 38] and vice versa. If these interactions are altered by environmental drivers such as air pressure, cascading consequences for ecosystem functioning may arise [39].

We therefore used the terraXcube Ecotron facility, which can simulate varying climatic conditions, to investigate the effects of moderately altered air pressure on rhizosphere microorganisms associated with three plant species in terms of microbial diversity, community composition, activity and biomass. We hypothesize that (i) reduced air pressure leads to decreased microbial biomass, activity and microbial diversity in the plant rhizosphere; (ii) the observed microbial responses to air pressure are not driven by plant morphological and physiological traits; and (iii) rhizosphere communities of different plant species respond divergently to reduced air pressure.

## Material and methods

### Study site description and sample collection

Plant individuals and soil used for the experimental setup were collected from the Long-Term Socio-Ecological Research (LTSER) site ‘Val Mazia/Matschertal’ in South Tyrol, the northernmost province of Italy (LTER_EU_IT_097, 46.6928°N, 10.6157°E). The sampling site is a perennial montane grassland (*Festucetum valesiacae*) located at 1500 m a.s.l. The Val Mazia/Matschertal catchment is a relatively dry Inner-Alpine valley spanning over 90 km, with elevations ranging from 1000 to 3700 m a.s.l., and is well described in terms of above- and belowground biodiversity [40, 41]. In May 2022, at the onset of the growing season, 40 individuals each of three frequently co-occurring plant species, namely *Brachypodium rupestre* (a grass), *Hieracium pilosella* (a forb), and *Trifolium pratense* (a legume), were collected at a similar phenological stage (with unfolded leaves but no visible inflorescence) by extracting soil–plant plugs (4.8 cm^2^ × 7.5 cm). From the same area, bulk soil was sampled from the upper mineral horizon (10–20 cm depth) after removing the vegetation cover. Plants and soil were immediately transported to the laboratory, where the soil was sieved (< 4 mm) and thoroughly homogenized. All plant plugs were stored at 15 °C for 24 h prior to transplantation.

### Experimental setup

To assess the impact of atmospheric pressure on rhizosphere microorganisms, we used hypobaric chambers (terraXcube) to simulate four elevational levels [m a.s.l.] by adjusting the air pressure to (i) 98 kPa (corresponding to 260 m a.s.l.), (ii) 85 kPa (1500 m a.s.l.), (iii) 75 kPa (2500 m a.s.l.), and (iv) 62 kPa (4000 m a.s.l.). To disentangle effects of atmospheric pressure from other physical parameters that typically covary with elevation, all chambers were kept under identical conditions of temperature, relative humidity, and solar radiation. These values were derived from field records of a climate station located at the 1500 m LTSER site. Average values for the incubation period were 18 °C, 43% relative humidity, and 620 μmol m^−2^ s^−1^ photosynthetically active radiation. Temperature, humidity, and light conditions were varied on an hourly basis to reproduce natural daily cycles (Supplementary Figure S1). The collected plant plugs were transplanted into 1.2 L pots (Ø 150 ^*^ height 180 mm) filled with 600 g of the sieved soil (4 mm, described above). For each simulated elevational level, 10 potted individuals of each species were placed in a single hypobaric chamber and incubated for 28 days. In total, four hypobaric chambers with different air pressure levels were maintained. To ensure an adequate water supply and prevent soil desiccation caused by elevation-related increases in VPD during incubation, pots were irrigated using an artificial irrigation system with UV-sterilized tap water. Irrigation was applied every 48 h for one minute with a flow rate of 2 L h^−1^, simulating summer field conditions at the plants’ site of origin [42]. Altogether, 120 pots were evenly distributed across the four hypobaric chambers. Within each chamber, 40 pots were arranged on two benches, with each bench containing an equal number of individuals from each plant species. Throughout the 28-day incubation period, both chamber and bench positions were systematically rotated to ensure uniform exposure to experimental conditions.

### Above-ground plant sampling, plant measurements and rhizosphere soil collection

After the incubation period, pots exhibiting poor plant growth were removed. To ensure a homogeneous sample set, two pots out of ten replicates with the poorest performing plants were removed per plant species, leaving eight out of ten pots for further analysis. Stomatal conductance (GS [mmol m^−2^ s^−1^]) and chlorophyll content (CHL [SPAD]) were measured on one top leaf of each plant by using a portable leaf porometer (SC-1, METER Group, Inc., Pullman, WA, USA) and a CCM-300 Chlorophyll Meter (Opti-Sciences, Hudson, NH, USA), respectively. The specific leaf area (SLA [mm^2^ mg^−1^]) was measured according to Pérez-Harguindeguy et al. [43] by randomly selecting fully expanded and photosynthetically active top leaves (per pot) of *T. pratense* (5 leaves), *H. pilosella* (1 leaf), and *B. rupestre* (2 leaves). Of these leaves, the leaf area (LA [cm^2^]) was determined using Image (https://imagej.net/ij/) and their fresh weight [g] and leaf dry weight [mg] (oven-dried at 70 °C for 72 h) were estimated. Using the leaf dry weight, the SLA and leaf dry matter content (LDMC) were calculated as follows: SLA = LA / leaf dry weight [cm^2^ mg^−1^]; LDMC = leaf dry weight / leaf fresh weight [mg g^−1^]. Aboveground biomass was collected and dried at 70 °C for 72 h to determine plant biomass [g]. Total carbon and nitrogen content of the oven-dried and homogenized aboveground plant biomass (Plant C_tot_ and Plant N_tot_) were measured with a CN analyzer (Truspec CHN Macro, Leco, MI, United States). Rhizosphere samples were subsequently collected individually from the corresponding pots by first shaking off bulk soil and any loosely attached soil by gentle handshaking and then removing the soil still adhering to the root surface (rhizosphere soil) of the entire root system (approx. 2 mm). This tightly attached soil was carefully detached from the root system using sterile forceps and tweezers. Per pot, the rhizosphere soil from one complete root system was collected, yielding in approx. 20–50 g of fresh soil, which was stored at 4 °C. The roots were then carefully rinsed with tap water and oven-dried at 70 °C for 72 h to determine root biomass [g]. To account for the small amount of rhizosphere soil, two individual replicates from different benches were pooled to form one composite sample. This resulted in a total of four replicate samples per plant species and air pressure level. The collected soil was sieved to 2 mm. Soil samples were stored at 4 °C for soil property analyses and frozen at − 20 °C for microbial and molecular analyses.

### Physico-chemical soil properties, microbial biomass and microbial activity

Dry weight (dw [g g^−1^ soil]) was determined gravimetrically by drying 5.0 g of soil at 105 °C overnight. Oven-dried soil was analyzed on a CN analyzer (Truspec CHN Macro, Leco, MI, United States) to estimate the total carbon (C_tot_ [%]) and total nitrogen (N_tot_ [%]) contents. The determination of soil pH was done electrochemically in a 1:2.5 (w/v) soil:CaCl_2_ solution [0.01 M] at room temperature. Plant-available ammonium (NH_4_^+^-N [µg g^−1^ dw]) and nitrate (NO_3_^−^-N [µg g^−1^ dw]) were measured on a San^++^ Continuous-Flow Analyser (Skalar, Netherlands). Microbial biomass carbon (MBC [mg C g^−1^ dw]) was determined using the chloroform fumigation-extraction technique: 3.0 g of soil was fumigated with chloroform for 24 h and extracted in a 1:5 (w/v) soil:K_2_SO_4_ solution [0.5 M]. The dehydrogenase activity (DHA [µg TPF g^−1^ dw 16 h^−1^]) was estimated by measuring triphenyl formazan photometrically at 546 nm, after adding the substrate triphenyltetrazolium chloride (TTC) to 1.0 g of soil [44].

### DNA extraction and amplicon sequencing of prokaryote and fungal communities

Total genomic DNA was extracted from 200 mg rhizosphere soil using a QIAcube Connect (Qiagen, Germany) with the DNeasy® PowerSoil® Pro Kit (Qiagen, Germany) following the manufacturer’s recommendations. Extraction controls to detect contamination during DNA extraction (lysis buffer only, no sample material) and positive controls to validate the sequencing process (ZymoBIOMICS™ Microbial Community Standard, Cat. No D6300) were also extracted. The quantity and quality of all DNA extracts was evaluated via UV/VIS spectrophotometry on a NanoDrop 2000cTM (PeqLab, Germany). DNA extracts were submitted to Novogene (UK) for Illumina NovaSeq 6000 sequencing with a paired-end approach (250 bp). The V3-V4 region of the prokaryote 16S rRNA gene (primer pair 341F/805R, [45, 46]) and the fungal ITS2 region (primer pair ITS3-2024F/ITS4-2409R [47]) were targeted. Non-template controls to detect contamination during sequencing (water only, no DNA extraction), extraction controls and positive controls were also sequenced.

### Prokaryote 16S rRNA and fungal ITS2 downstream analysis

Primer free, paired-end reads of the 16S rRNA gene were processed following the 1.16 DADA2 tutorial workflow (https://benjjneb.github.io/dada2/tutorial.html), using the DADA2 package [48] in R (v 4.2.0, [49]). Reads were quality filtered using the *filterAndtrim* function applying the following parameters: truncLen = c(220, 220), maxN = 0, maxEE = c(1,2), truncQ = 2. ASVs were generated using the DADA2 inference algorithm by the *learnErrors* and *dada* functions. Reads were merged (*mergePairs* function) and chimeric sequences were removed (*removeBimeraDenovo* function). The taxonomy was assigned (*assignTaxonomy* function) using the SILVA SSU 138.1 database [50] and the RDP Naive Bayesian Classifier algorithm [51].

For fungi, the ITS2 subregion was extracted using the ITSx open source software utility [52]. With the extracted sequences, OTUs were constructed applying a 97% similarity threshold. OTU-clustering was preferred over the ASV-approach to account for the high variability of the ITS2 region [53]. For processing, mothur (v.1.48.0) [54] was used, following the protocol outlined in Rzehak et al. [53], with two specific settings: (i) for the *make.contigs* function, the following parameters were used: maxhomop = 9, maxambig = 0, insert = 20, and deltaq = 6; (ii) within the *classify.seqs* function, the bootstrap cutoff for taxonomy assignment was set to 60%.

Further processing of prokaryote (16S rRNA, inferred ASVs) and fungal (ITS2 region, generated OTUs) sequencing results was conducted in R [49]. Contaminant OTUs/ASVs were identified using the *decontam* package [55] by comparing the microbial community of the soil samples with that of extraction and non-template controls. Identified contaminants were removed from the OTU/ASV tables. Removal of rare OTUs/ASVs was done by applying a relative abundance threshold (according to Rzehak et al. [53]), which was defined by the relative abundance of 5 reads in relation to the mean total read count across samples. Reads with a relative abundance of less than 0.0064% for prokaryotes and less than 0.0068% for fungi were defined as rare and excluded from the dataset. On average, single libraries contained 73,442 reads (prokaryote libraries) and 78,021 reads (fungal libraries). Libraries ranged from 36,403 to 83,858 (prokaryote libraries) and 25,432 to 98,776 reads (fungal libraries), while the median sequencing depth per sample was 74,356 reads for prokaryotes and 85,391 reads for fungi, respectively (Supplementary Table S1). To remove biase introduced by different library sizes, cumulative sum scaling in combination with log transformation (CSS normalization) of read counts was conducted using the *metagenomeSeq* package [56].

### Data analysis

All statistical analyses and plots were performed in R [49]. Significant differences in environmental properties (soil chemistry, microbial biomass and activity, morphological and physiological plant traits) among plant species were tested using one-way or multifactorial ANOVA, followed by Fisher’s Least Significant Difference (LSD) post-hoc test. Linear relationships between environmental properties and air pressure levels were assessed using general linear models (GLMs) and differences of slopes between plant species were identified by a GLM testing homogeneity of slopes. Scatterplots displaying the GLM regression fits alongside data points were generated using *ggplot2* [57]. Microbial community data was processed using the packages *microeco* [58], *phyloseq* [59], and *vegan* [60]. Microbial diversity, given as alpha and beta diversity of prokaryotes and fungi, was analyzed to assess the differences in rhizosphere communities of three plant species incubated under varying air pressure levels. Microbial alpha diversity was measured using observed species richness (OTUs or ASVs) and the Shannon Index. Significant differences in microbial alpha diversity across plant species were tested via a one-way or multifactorial ANOVA followed by Fisher’s Least Significant Difference (LSD) post-hoc test. Additionally, relationships between alpha diversity and air pressure were evaluated using GLMs. Bray–Curtis dissimilarities were calculated from CSS-normalized OTU/ASV-tables, followed by non-metric multidimensional scaling (NMDS) ordination to visualize the overall structure of the data and highlight sample patterns. Significant differences in microbial community composition among plant species and air pressure levels were tested using PERMANOVA with 999 permutations. Differential abundance analyses of prokaryote and fungal genera across plant species and pressure levels were performed using ALDEx2, applying the Kruskal–Wallis test. Genera with a Benjamini–Hochberg corrected *p* value < 0.05 were identified as biomarker genera specific to a particular plant species or air pressure level.

Linear relationships between environmental variables (air pressure, soil properties, microbial biomass, morphological and physiological plant traits) and microbial communities (CSS-normalized read counts) in the rhizosphere soil were examined using redundancy analysis (RDA) with 999 permutations, conducted separately for each plant species. Prior to RDA, multicollinearity among environmental variables was assessed by (i) calculating Pearson correlation coefficients, (ii) estimating Variance Inflation Factors (VIF), and (iii) performing Principal Component Analyses (PCA). Due to collinearity with other variables (VIF > 7), N_tot_, Plant_N_tot_, SLA, and LDMC were excluded from the analysis. RDA results were presented in a table displaying the sum of squares, F-values and p-values. The relationships between microbial community structures and explanatory variables were visualized in a multivariate space, with community data represented as points and significant environmental variables as vectors.

## Results

### Physico-chemical soil properties, microbial biomass, microbial activity, and root biomass

All physico-chemical soil properties differed significantly across plant species (ANOVA, p < 0.05), as did all plant trait measurements except chlorophyll content (Supplementary Table S2). Overall, *T. pratense* exhibited the highest plant biomass, as well as the highest microbial biomass carbon and activity within the rhizosphere, followed by *B. rupestre* and *H. pilosella* (Supplementary Table S2). Microbial biomass and microbial activity were significantly affected by air pressure in a plant-specific manner (*p* < 0.01).

**Fig. 1:**
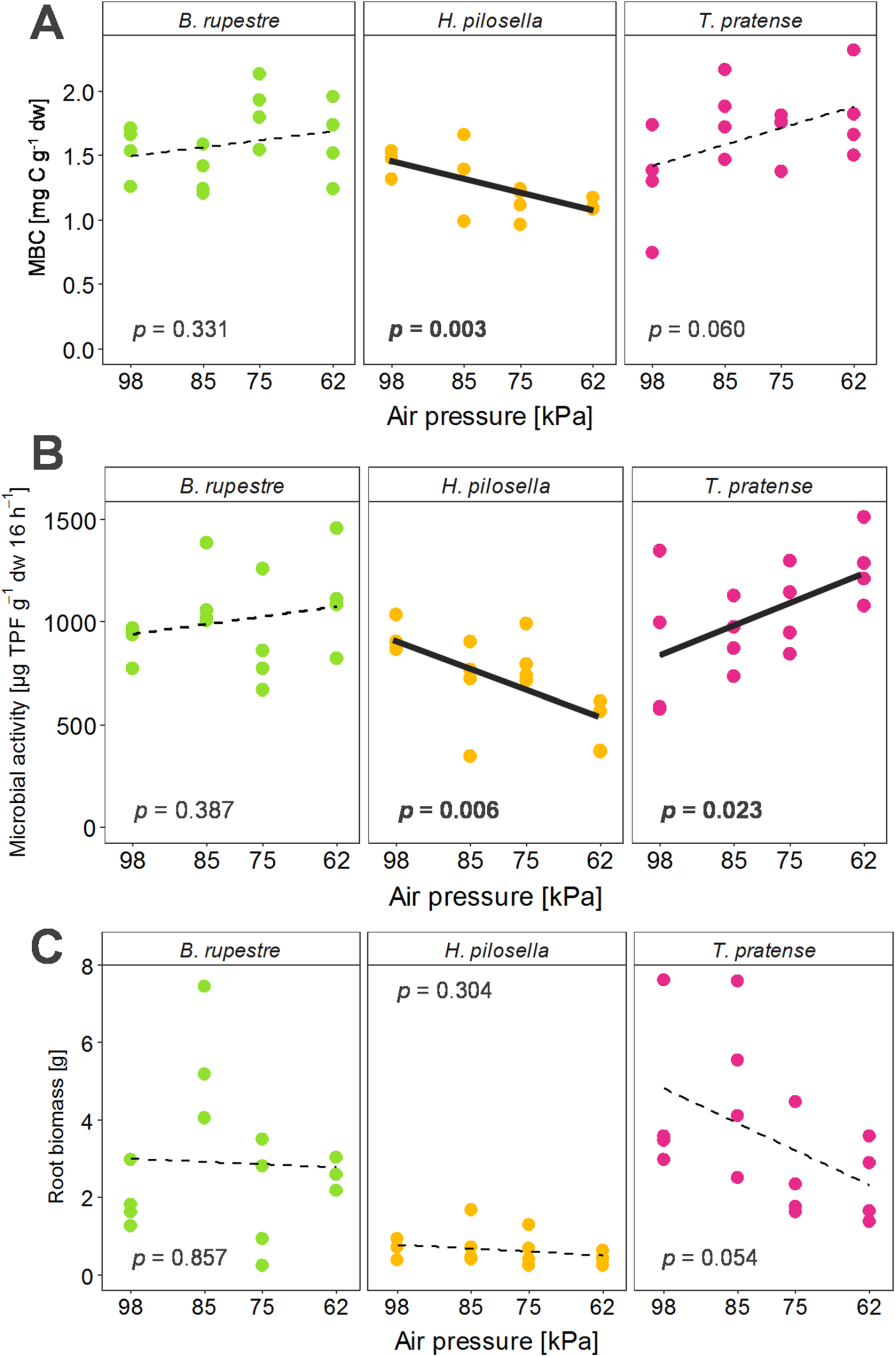
Microbial biomass carbon (**A**) and microbial activity (**B**) in the rhizosphere, and root biomass (**C**) of *B. rupestre*, *H. pilosella*, and *T. pratense* after growth at different air pressure levels (98, 85, 75, 62 kPa). Individual data points are shown along with dashed lines representing separate linear regression fits. Associated *p-*values report the significance of the relationships in each plot. Significant relationships (*p* < 0.05) are indicated by bold *p*-values and solid regression lines. Slopes of microbial biomass carbon and microbial activity differed significantly among plant species (*p* < 0.01)

In the rhizosphere of *B. rupestre* and *T. pratense*, microbial biomass and microbial activity showed an increase from high to low air pressure (98 – 62 kPa), with a significant increase in microbial activity observed for *T. pratense* (Fig. 1A, B; Supplementary Table S2). In contrast, *H. pilosella* exhibited a significant decrease in both microbial biomass and activity with decreasing air pressure (Fig. 1A, B)*.* Root biomass was not significantly affected by air pressure in any plant species and showed no consistent trend (Fig. 1C; Supplementary Table S3).

### Microbial alpha diversity

Both observed species and the Shannon Index exhibited slight, non-significant variation along the air pressure gradient and followed plant species-specific patterns (Supplementary Figures S2 and S3). In the rhizosphere of *B. rupestre*, the mean number of observed prokaryote species was 1,816, but no consistent trend was observed across air pressures of 98, 85, and 75 kPa. At the lowest air pressure (62 kPa), this number slightly increased to 1,957 (Supplementary Figure S2A). A similar trend was observed in the fungal community of *B. rupestre,* where the mean number of observed species was 452 and slightly increased to 508 at 62 kPa (Supplementary Figure S2B). In contrast, the prokaryote community in the rhizosphere of *H. pilosella* showed a slight decrease in observed species with decreasing air pressure, from 1,902 species at 98 kPa to 1,757 at 62 kPa (Supplementary Figure S2A). The fungal community of *H. pilosella* did not exhibit a clear response to air pressure changes, with a mean of 511 observed species across all pressure levels (Supplementary Figure S2B). For *T. pratense*, neither the prokaryote nor the fungal communities showed a consistent pattern with air pressure. The number of observed prokaryote species ranged between 1,773 to 1,807, and fungal species from 478 to 420, between 98 and 62 kPa air pressure (Supplementary Figure S2). Changes in Shannon Index values for both prokaryote and fungal communities mirrored those in the number of observed species along the air pressure gradient (Supplementary Figure S3).

### Microbial community structure in relation to environmental variables

Across all plant species (*B. rupestre*, *H. pilosella* and *T. pratense*), the composition of both prokaryote and fungal communities in the rhizosphere differed significantly under control conditions, where plants and soil were exposed to air pressure occurring at their natural habitat (1500 m a.s.l., corresponding to 85 kPa air pressure) (PERMANOVA, *p* < 0.001; Supplementary Figure S4A, B). When the air pressure was changed, prokaryote community composition in the rhizosphere of all plants species varied significantly (PERMANOVA, *p* < 0.01; Supplementary Table S4).

**Fig. 2:**
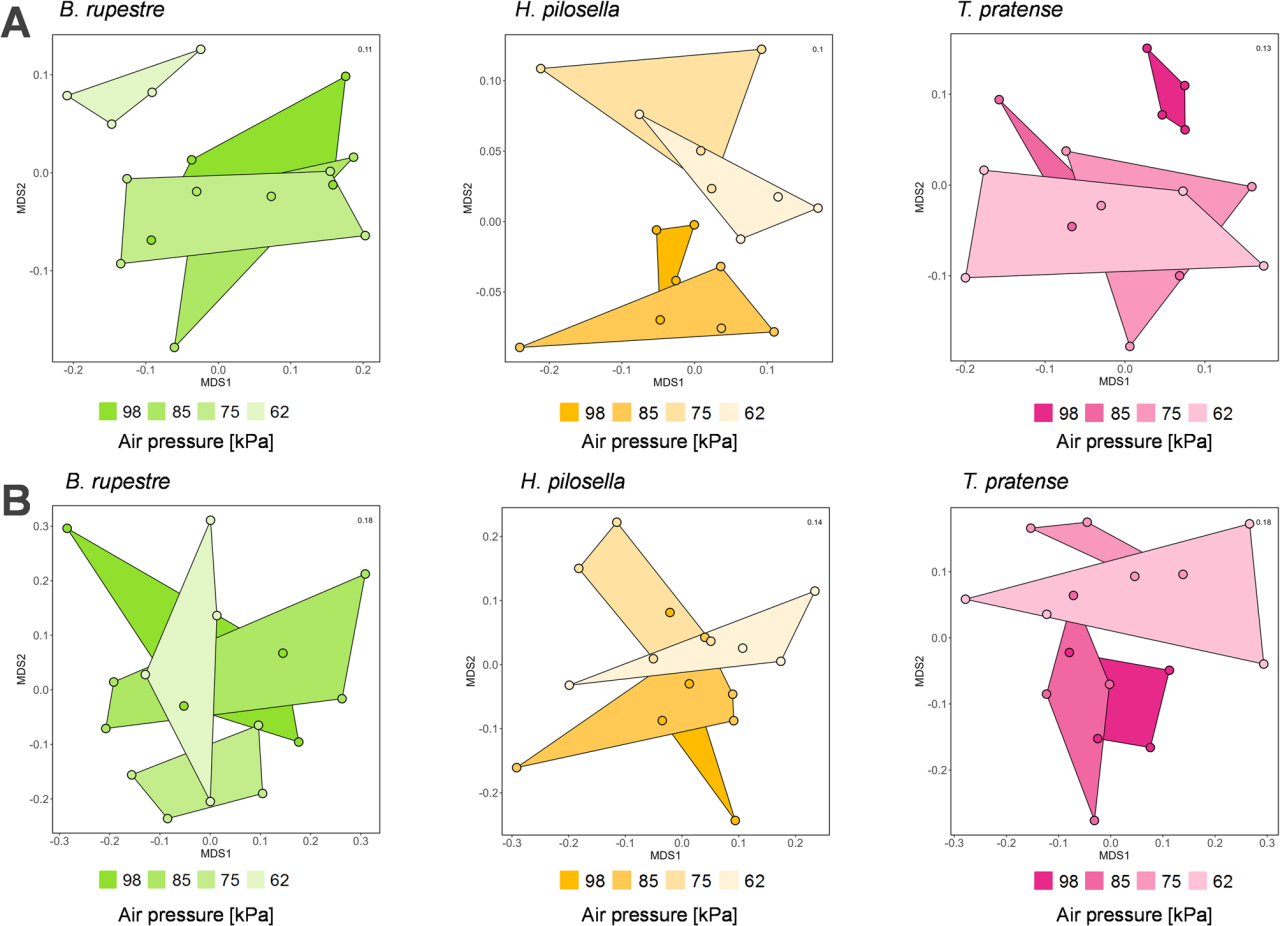
Prokaryote (**A**) and fungal (**B**) community structure represented by NMDS-plots based on Bray–Curtis dissimilarities for different plant species (*B. rupestre*, *H. pilosella*, *T. pratense*). Plant species are indicated by different colors and shades represent different air pressure levels (98, 85, 75, 62 kPa). The stress value (shown in the top right corner of the plots) indicates the goodness of fit of the ordinations

In *B. rupestre*, NMDS plots based on Bray–Curtis dissimilarities showed overlapping convex hulls for samples incubated at 98, 85 and 75 kPa, indicating that prokaryotic community composition remained largely consistent across these air pressure conditions (Fig. 2A). In contrast, microbial communities from 62 kPa formed a distinct, non-overlapping cluster, suggesting a compositional shift at lower air pressure (62 kPa) (Fig. 2A). For *H. pilosella,* rhizosphere samples clustered into two groups: one comprising high air pressure levels (98 and 85 kPa) and the other low air pressure levels (75 and 62 kPa) (Fig. 2A). In *T. pratense*, samples incubated at the highest air pressure (98 kPa) formed a separate cluster, with no overlap with samples from 85, 75, or 62 kPa (Fig. 2A), again indicating a distinct community structure at this pressure. In contrast to the prokaryotes, fungal communities in the rhizosphere of *B. rupestre* and *H. pilosella* did not differ significantly with air pressure (PERMANOVA, *p* > 0.05; Supplementary Table S4; Fig. 2B). However, in *T. pratense*, fungal community composition differed significantly between high (98, 85 kPa) and low (75, 62 kPa) air pressures (PERMANOVA, *p* < 0.05; Fig. 2B; Supplementary Table S4).

**Fig. 3:**
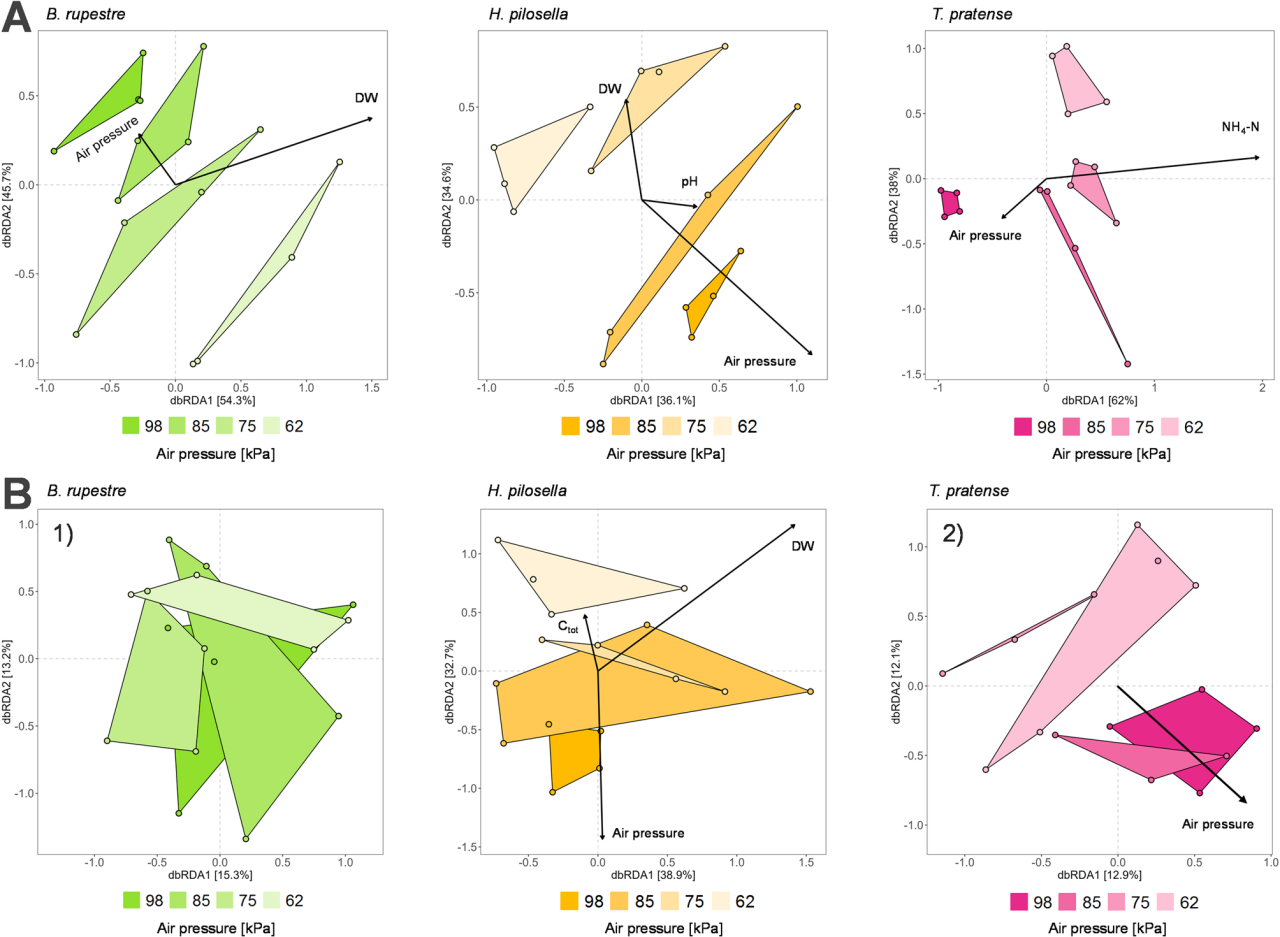
Ordination plots representing the results of the redundancy analysis (RDA). Samples representing prokaryote (**A**) and fungal (**B**) community compositions are displayed as points positioned along the ordination axes. Explanatory environmental variables are represented as arrows with their direction indicating the gradient of increasing values. Only environmental predictors significantly contributing to the explanation of microbial community variance are shown. The proportion of variance explained by each axis is indicated in brackets. RDA results are plotted separately for different plant species (*B. rupestre*, *H. pilosella*, *T. pratense*). Plant species are indicated by different colors and shades represent different air pressure levels (98, 85, 75, 62 kPa). In the number-marked plots, the original RDA including all environmental variables is shown. In plot 1), no arrows are included because none of the variables were statistically significant. In contrast, plot 2) displays only the single significant environmental variable as an arrow

Redundancy analysis (RDA) was conducted to explore the relationships between environmental variables (air pressure, soil properties, microbial biomass, morphological and physiological plant traits) and the composition of prokaryote and fungal communities in the rhizosphere (Fig. 3). The analysis showed that air pressure significantly influenced prokaryote community composition across all plant species (Table 1, *p* < 0.05). In the RDA ordination plots with only significant variables (Fig. 3A), the direction of the arrow representing the air pressure effect aligned with the observed community shifts: in *B. rupestre*, *H. pilosella* and in *T. pratense* all air pressure levels were distinctly separated from each other. In addition to air pressure, other significant, plant-specific predictors of prokaryote community variation included dry weight (*B. rupestre* and *H. pilosella*), pH (*H. pilosella*) and NH_4_^+^-N (*T. pratense*). For fungal communities, air pressure significantly influenced the community composition in *H. pilosella* and *T. pratense,* but not in *B. rupestre* (Fig. 3B; Table 1).

**Table 1:** Results of a permutation test for explanatory variables, following a redundancy analysis (RDA)

	Plant species	Variable	SumOfSqs	F	*p*-value	Significance
Prokaryotes	*B. rupestre*	Air pressure	0.111	1.255	0.013	*
		DW	0.114	1.289	0.007	**
		pH	0.099	1.119	0.11	
		NH_4_^+^-N	0.095	1.068	0.228	
		NO_3_^−^-N	0.099	1.120	0.118	
		MBC	0.086	0.970	0.606	
		C_tot_	0.099	1.120	0.111	
		Plant_biomass	0.094	1.058	0.242	
		Plant_C_tot_	0.094	1.061	0.248	
		GS	0.095	1.075	0.206	
		CHL	0.102	1.152	0.091	
		Root_biomass	0.088	0.999	0.453	
		Residual	0.265			
	*H. pilosella*	Air pressure	0.116	1.325	0.005	**
		DW	0.107	1.220	0.025	*
		pH	0.104	1.193	0.042	*
		NH_4_^+^-N	0.099	1.125	0.094	
		NO_3_^−^-N	0.096	1.092	0.182	
		MBC	0.095	1.086	0.179	
		C_tot_	0.097	1.103	0.15	
		Plant_biomass	0.083	0.947	0.707	
		Plant_C_tot_	0.087	0.997	0.458	
		GS	0.088	1.001	0.454	
		CHL	0.080	0.918	0.807	
		Root_biomass	0.088	1.000	0.464	
		Residual	0.263			
	*T. pratense*	Air pressure	0.134	1.532	0.001	***
		DW	0.079	0.904	0.83	
		pH	0.098	1.123	0.161	
		NH_4_^+^-N	0.110	1.263	0.029	*
		NO_3_^−^-N	0.101	1.159	0.095	
		MBC	0.097	1.112	0.162	
		C_tot_	0.099	1.128	0.124	
		Plant_biomass	0.099	1.131	0.133	
		Plant_C_tot_	0.089	1.018	0.395	
		GS	0.085	0.968	0.62	
		CHL	0.100	1.145	0.103	
		Root_biomass	0.089	1.023	0.404	
		Residual	0.175			
Fungi	*B. rupestre*	Air pressure	0.150	1.052	0.367	
		DW	0.174	1.219	0.139	
		pH	0.168	1.179	0.196	
		NH_4_^+^-N	0.169	1.185	0.194	
		NO_3_^−^-N	0.187	1.307	0.083	
		MBC	0.158	1.107	0.291	
		C_tot_	0.141	0.986	0.492	
		Plant_biomass	0.142	0.993	0.473	
		Plant_C_tot_	0.167	1.170	0.203	
		GS	0.141	0.989	0.49	
		CHL	0.121	0.847	0.793	
		Root_biomass	0.104	0.731	0.946	
		Residual	0.428			
	*H. pilosella*	Air pressure	0.136	1.363	0.042	*
		DW	0.164	1.642	0.006	**
		pH	0.130	1.302	0.066	
		NH_4_^+^-N	0.120	1.202	0.123	
		NO_3_^−^-N	0.101	1.016	0.442	
		MBC	0.134	1.342	0.051	
		C_tot_	0.135	1.357	0.037	*
		Plant_biomass	0.119	1.196	0.138	
		Plant_C_tot_	0.100	1.002	0.472	
		GS	0.107	1.077	0.31	
		CHL	0.098	0.979	0.497	
		Root_biomass	0.096	0.960	0.548	
		Residual	0.299			
	*T. pratense*	Air pressure	0.184	1.380	0.013	*
		DW	0.135	1.012	0.449	
		pH	0.142	1.069	0.307	
		NH_4_^+^-N	0.137	1.031	0.41	
		NO_3_^−^-N	0.155	1.164	0.121	
		MBC	0.129	0.969	0.596	
		C_tot_	0.143	1.077	0.304	
		Plant_biomass	0.133	1.000	0.494	
		Plant_C_tot_	0.114	0.857	0.889	
		GS	0.123	0.928	0.706	
		CHL	0.140	1.051	0.363	
		Root_biomass	0.159	1.194	0.094	
		Residual	0.266			

The table presents the sum of squares (SumOfSqs), F-values (F), and p-values for each explanatory variable. Asterisks (*) indicate statistically significant effects (*** for *p* < 0.001, ** for *p* < 0.01, * for *p* < 0.05). The explanatory variables are air pressure, rhizosphere soil properties: dry weight (DW), pH, ammonium (NH_4_^+^-N), nitrate (NO_3_^−^-N), microbial biomass carbon (MBC), total carbon (C_tot_) and plant properties: Plant_biomass, plant total carbon (Plant_C_tot_), stomatal conductance (GS), chlorophyll content (CHL), Root_biomass.

In the corresponding RDA ordination plots (Fig. 3B), the air pressure vector indicated separation of the lowest air pressure level (62 kPa) from others in *H. pilosella*, and a clear split between high (98, 85 kPa) and low-pressure levels (75, 62 kPa) in *T. pratense*. No clear separation was observed in *B. rupestre*. Additional significant, plant-specific predictors of fungal community variation included DW and C_tot_ (*H. pilosella*). Notably, none of the determined morphological or physiological plant traits included in the RDA (plant biomass, root biomass, Plant C_tot_, GS, CHL) were significant predictors of prokaryote or fungal community composition in the rhizosphere soils of any plant species studied (Table 1).

### Prokaryote and fungal biomarkers for air pressure levels and plant species

The most abundant prokaryote orders across the rhizospheres of all plant species were Bacillales, Rhizobiales and Solirubrobacterales (Supplementary Figure S5A). Dominant fungal orders included Hypocreales, Pleosporales and Agaricales (Supplementary Figure S5B). No prokaryote or fungal genus was significantly associated with any single air pressure condition (98, 85, 75, 62 kPa) across plant species. However, several microbial biomarkers were identified as being significantly associated with specific plant species (ALDEx analysis on genus level, *p* < 0.05). In total, several prokaryote and fungal biomarker genera were identified for the three plant species (*B. rupestre*, *H. pilosella*, and *T. pratense*), with a subset of these being highly significant (*p* < 0.001). The relative abundances of the highly significant biomarkers across plant species are shown in Supplementary Figure S6. For *B. rupestre*, five highly significant prokaryote biomarkers were identified, representing different orders such as Gemmatales and Rubrobacterales. The genus *Nocardia* (order Corynebacteriales) was more associated with *H. pilosella*, while *Geobacter* (order Geobacterales) was characteristic for *T. pratense*. Regarding fungi, several highly significant biomarker genera (*p* < 0.001) were identified for the three plant species, representing diverse fungal orders. For *B. rupestre*, biomarkers were associated with the orders Pleosporales, Chaetothyriales, Helotiales, and Hypocreales. *H. pilosella* was linked to biomarkers from the orders Pleosporales, Venturiales, Chaetothyriales, Sebacinales, and Tremellales. For *T. pratense*, fungal biomarkers were classified within the orders Capnodiales and Hypocreales. A full list of identified biomarkers is provided in Supplementary Table S5.

## Discussion

In this study, we conducted an experiment in hypobaric Ecotron chambers (terraXcube) to investigate how changes in air pressure affect biomass, activity and diversity of rhizosphere microorganisms associated with three plant species (*B. rupestre*, *H. pilosella*, and *T. pratense*), presumably migrating to higher elevations to evade rising temperatures during climate change.

### Air pressure affects biomass and activity of rhizosphere microorganisms

We observed plant-specific, pressure-related changes in microbial activity and biomass. In line with our hypothesis that a reduced air pressure would decrease the activity of rhizosphere microorganisms, we could establish a reduced microbial activity in the rhizosphere of *H. pilosella* with decreasing air pressure. In contrast, activity significantly increased in the rhizosphere of *T. pratense* and not significantly in *B. rupestre* rhizosphere. Even when indirect effects of air pressure such as desiccation and decreased gas concentration are controlled for, pressure was shown to directly affect microbial cells by altering cell volume (causing shrinkage or swelling), modifying membrane fluidity, and impacting protein folding as well as enzyme reaction kinetics [29, 61]. And depending e.g., on their cell structure and executed biochemical pathways, microorganisms vary in their responses to pressure [62]. We determined significantly different compositions of microorganisms in the rhizosphere of the three plant species (*B. rupestre, H. pilosella, T. pratense*), likely driven by species-specific exudate profiles, which differ among host plants [63]. The community differences among rhizospheres indicate the presence of distinct physiological strategies and cell structures of microorganisms; therefore, varying responses of different communities to the same environmental parameter are plausible. For example, gram-positive bacteria tend to react differently to pressure than gram-negative bacteria [62] and indeed, the majority of bacterial genera most strongly associated with *T. pratense* were gram-negative, while numbers of gram-positive and gram-negative bacterial biomarker genera were balanced in *B. rupestre* and *H. pilosella* rhizospheres, pointing to different distributions of cell structures among rhizospheres of plant species and thus might contribute to different responses to air pressure. Furthermore, we found evidence suggesting that the presence of specific physiological pathways, reflected in distinct enzyme repertoires within microbial cells, differed among rhizosphere communities. For example, the prokaryote genus *Geobacter*, which can accelerate CH_4_ production as a key syntroph and is ecologically versatile [64], was most strongly associated with *T. pratense*, while the prokaryote genus *Nocardia*, an opportunistic pathogen producing various antimicrobial secondary metabolites [65], was associated with *H. pilosella*. Furthermore, the fungal genus *Periconia*, which exhibits activity against phytopathogenic oomycetes and fungi, and phytophagous nematodes [66], was strongly associated with *B. rupestre*. Hence, plant-specific pressure responses may have resulted from the specific microorganisms (exhibiting specific biochemical pathways and cell structures) prevailing in the rhizospheres of different plant species. It has been demonstrated that pressure can influence a broad range of enzyme-catalyzed reactions in the range of kilobar-changes [67] and that enzymes of microbial species vary significantly in their ability to tolerate pressure [62]. This suggests that enzymatic function and thereby metabolic activity of rhizosphere microorganisms may be directly affected by changed air pressure and explains the significant, pressure-related changes of microbial activity in *H. pilosella* and *T. pratense*. In a laboratory study it was shown that *B. subtilis* cells upregulated many genes, usually highly expressed in response to environmental stresses like heat and cold, when low pressure conditions (50 mbar, corresponding to 5 kPa) were applied [33]. Lower air pressure may have induced such a stress response in the distinct microbial community of *H. pilosella*, leading to hampered cell growth and cell metabolism [68] and resulting in a significantly lowered microbial activity. Furthermore, studies concluded that growth rate and yield of bacterial cells can be inhibited by low air pressure [30], which could explain the significantly reduced microbial biomass in *H. pilosella* under lower air pressure. In contrast, the increased gas exchange under lower air pressure conditions [29] may have supported the cell metabolism of the specific microorganisms associated with *T. pratense*, resulting in significantly increased microbial activity. However, studies exposing pure cultures of mesophilic microorganisms to low air pressure predicted a low-pressure limit of 100 mbar (corresponding to 10 kPa) for microorganisms, above which growth of microorganisms may not be affected [33]. This low-pressure limit corresponds to an elevation of 18 km and is much lower than the air pressure change conducted in the current study (98 to 62 kPa). Generally, current knowledge about air pressure responses of microorganisms is based on microorganisms inhabiting environments with extremely high or low pressure (hyperbaric or piezophilic microorganisms in deep sea and microorganisms in space) and very little is known about how microorganisms respond to a moderate reduction of pressure [29, 33]. Furthermore, our study focused on microbial communities under near-natural conditions, in contrast to many previous studies that examined microbial pressure responses in pure cultures under optimal growth conditions [29]. Such conditions may elicit markedly different responses from those of a complex rhizosphere community.

### Plant-specific rhizosphere responses to air pressure variation

We observed that altering air pressure to higher or lower levels than the level typical for the natural habitat of the study plants (1500 m a.s.l.) significantly affected the rhizosphere microorganisms. While changes in air pressure did not significantly influence the alpha diversity of rhizosphere microorganisms associated with *B. rupestre*, *H. pilosella* and *T. pratense*, they did lead to significant shifts in microbial community composition across all plant species. This pattern was also observed for environmental stressors such as drought, which altered community composition but did not affect alpha diversity [16]. We can confidently exclude that a shift of microbial community composition is due to other incubation parameters (e.g., temperature, moisture), as incubated plant pots of all hypobaric chambers experienced exactly the same, constant conditions, and thus, observed community shifts should be attributable to air pressure. Additionally, all pots were filled with the same homogenized, sieved soil, ensuring a uniform starting microbial community and substrate environment.

Abiotic stressors such as drought [16, 69] and soil pH [70] are known to alter rhizosphere communities, with potential consequences for ecosystem processes [71], although such compositional changes are not always reflected in alpha diversity [16]. In contrast, air pressure has not previously been considered as a driver of rhizosphere microbial dynamics. Given that both drought and reduced air pressure can influence gas exchange, changes in air pressure may similarly affect below-ground carbon allocation and, consequently, rhizosphere community composition [72]. It thus cannot be ruled out that reduced photosynthetic carbon input under low air pressure limits root exudation and alters the turnover of recently assimilated carbon, with the magnitude of these effects depending on stress intensity, as shown for drought by Oram et al. [72]. Conversely, Enderle et al. [73] found that drought-induced changes in rhizosphere communities indirectly affected plant growth. However, the community shifts observed here may thus reflect differential adaptation of microbial taxa to altered pressure induced changes in available carbon. Supporting this idea other drought studies have reported an enrichment of Actinobacteria and Firmicutes [69, 74]. In contrast, pressure-related community shifts in our study could not be attributed to specific microbial taxa, suggesting that structural changes may have occurred at a finer taxonomic or functional level, potentially reflecting functional redundancy or shared adaptive traits across phylogenetically diverse groups.

Alternatively, the observed shifts in microbial community composition may result from direct or indirect effects of air pressure-induced changes in plant traits. For instance, drought-related alterations in the root microbiome of plants were found to be indirectly mediated by host plant responses to drought [16]. Interestingly, we could not detect a consistent pattern of microbial community separation across air pressure levels. Instead, the composition of prokaryotes and in some cases, fungi varied with air pressure in a plant species-specific manner, indicating distinct pressure-related shifts across different rhizospheres. We could prove that community composition varies considerably within the rhizosphere of different plant species [11, 69, 75]. These findings highlight that even in standardized soil, the rhizosphere microbiota remains highly plant-specific, with potential consequences for plant–microbe interactions and functional traits in multi-species systems. Species-specific communities are likely shaped by divergent exudate profiles, as plants modulate rhizosphere microbial composition and activity via root exudation, which alters both carbon availability and physicochemical conditions [12, 76]. These significantly different rhizosphere communities might have served as distinct starting point for direct or indirect effects of air pressure-induced changes in plant traits. Plants suffering abiotic stresses can actively modulate their rhizosphere microbiome by altering both the quantity and composition of their root exudates [77] and microorganisms are actively recruited to the rhizosphere to improve the plant’s tolerance to environmental stressors [11, 75, 78, 79]. Indeed, the presence of stress-resistance-promoting microorganisms was shown to correlate with host plant gene expression and their excreted metabolites, such as amino acids and sugars [80]. In exchange for these nutritious root exudates, rhizosphere microorganisms support plant growth and stress resilience under abiotic pressures [9, 12]. But, the plant’s response to abiotic stress via root exudation differs fundamentally among various plant species and drives the response of their associated rhizosphere community to abiotic stress (e.g. drought) [81].

In the current study, we included plant species with contrasting traits and observed plant-specific community responses to changed air pressure. These patterns could be mediated by a plant-specific stress response of the host plants. By monitoring aboveground plant traits in the same experimental setup over one month, Lembo et al. [28] showed that reduced air pressure impacted plant eco-physiology (e.g.: stomatal conductance and chlorophyll content, but not growth parameters like dry matter production or height). Thus, initially we could not conclusively determine whether the observed microbial shifts found in our study were directly mediated by host plant responses to air pressure or pressure itself. However, none of the morphological and physiological plant traits (plant or root biomass, plant’s total carbon content, stomatal conductance, chlorophyll content) could be identified as a predictor of either prokaryote or fungal pressure-related shifts of community composition. This confirms our second hypothesis and suggests that responses of plant traits, such as plant and root biomass, plant’s total carbon content, stomatal conductance, chlorophyll content, to air pressure might play a minor role in shaping associated rhizosphere microorganisms. Instead, the observed changes in rhizosphere communities could be driven directly by air pressure itself. However, additional plant traits not measured in this study, such as root exudation, could also contribute to microbe-plant interactions in the rhizosphere and warrant further investigation. Moreover, there may be pressure related feedbacks of microorganisms on soil chemistry and plants, as, for example, soil C_tot_ or NH_4_^+^-N (Fig. 3) contents were in line with pressure-driven fungal and prokaryote community shifts.

Overall, we found that air pressure exerted a stronger impact on prokaryote than on fungal community composition. In all three plant species, prokaryote communities exhibited shifts in response to air pressure, and air pressure consistently emerged as a significant predictor of prokaryote community variation. In contrast, a significant pressure-related shift of the fungal community was observed only in *T. pratense*, and air pressure was identified as a significant predictor in *T. pratense* and *H. pilosella*, but not in the rhizosphere of *B. rupestre*. This is in line with previous findings that bacterial and fungal communities respond differently to environmental factors [41, 82–85] and that bacterial communities are often more sensitive to abiotic stressors such as drought than fungal communities [86]. Our findings therefore suggest that rhizosphere prokaryotes are more responsive to changes in air pressure than fungi, which could be due to their different growth rates or turnover times in soil, enormous differences of their size and owing to their different strategies for utilizing soil substrates [87, 88]. While all fungi have a quite simple heterotrophic metabolism, prokaryotes are capable of obtaining energy both phototrophically and chemotrophically, they use both organic and inorganic electron donors (organo- vs. lithotroph), and they can be both autotrophic and heterotrophic in terms of cellular carbon. As a result, changes in abiotic conditions can for example very quickly lead to an advantage for previously rare taxa and thus to a distinct change in prokaryote communities [89].

## Conclusion

Plants and their associated rhizosphere soils, collected from their native habitat at 1500 m a.s.l., were exposed to different air pressure levels using the innovative terraXcube Ecotron facility. In this controlled setup, elevation-related factors such as temperature, relative humidity, and solar radiation were kept constant to isolate the effects of air pressure. Microbial biomass and activity in the rhizosphere changed, with all effects being plant species-specific. Furthermore, the community composition of prokaryotes and, to a lesser extent, of fungi in the rhizosphere shifted in response to altered air pressure. Given that each plant species hosted distinct prokaryote and fungal communities characterized by unique biomarker genera, divergent responses of different rhizosphere communities to air pressure are plausible. Notably, air pressure, but none of the measured plant traits, was a significant predictor of microbial community structure. The lack of correlations between microbial community composition and the measured morphological and physiological plant traits suggests that microbial responses were independent of host plant traits but instead reflect a direct influence of air pressure on the rhizosphere microbiota. However, subtle feedbacks may still occur via microbial functional traits such as hormone modulation or altered antagonistic interactions-mechanisms that warrant further investigation.

As plants shift to higher elevations in response to ongoing climate change, they will inevitably encounter changes in their rhizosphere microbiota. The loss or replacement of key microbial taxa could lead to unpredictable consequences. Because rhizosphere communities are often finely tuned to specific plant species and local environmental conditions, these changes may disrupt existing plant–microbe relationships or introduce unfamiliar microbial partners with uncertain effects. Understanding whether such shifts ultimately benefit or harm soil chemistry, plant health and performance, and broader ecosystem functions remains a crucial question for future research.

## Supplementary Information

Below is the link to the electronic supplementary material.

Supplementary Material 1

Supplementary Material 2

## Data Availability

The sequencing data have been deposited in the NCBI Sequence Read Archive (SRA) under the BioProject ID PRJNA1277515. Additional raw data are available on Figshare under the following link: 10.6084/m9.figshare.29327183.v1.
