# Supplementary material for: Air pressure as a driver of plant-specific microbial responses in the rhizosphere
Source: Environ Microbiome. 2025 Nov 18;20:145. doi: 10.1186/s40793-025-00805-3 (PMC12625054; doi:10.1186/s40793-025-00805-3)
Supplement: Supplementary file 1 — Supplementary Material 1 [file 40793_2025_805_MOESM1_ESM.docx]

**Air pressure as a driver of plant-specific microbial responses in the rhizosphere**

Rzehak, Theresa^1*^; Praeg, Nadine^1^; Meul, Andreas^1^; Lembo, Silvia^1,2^; El Omari, Bouchra^2^; Dainese, Matteo^3^; Niedrist, Georg^2^; Illmer, Paul^1^

*^1^Department of Microbiology, Universität Innsbruck, Innsbruck, Austria*

*^2^Institute for Alpine Environment, EURAC Research, Bozen/Bolzano, Italy*

*^3^ Department of Biotechnology, University of Verona, Italy*

*corresponding author

**Supplementary Figures**


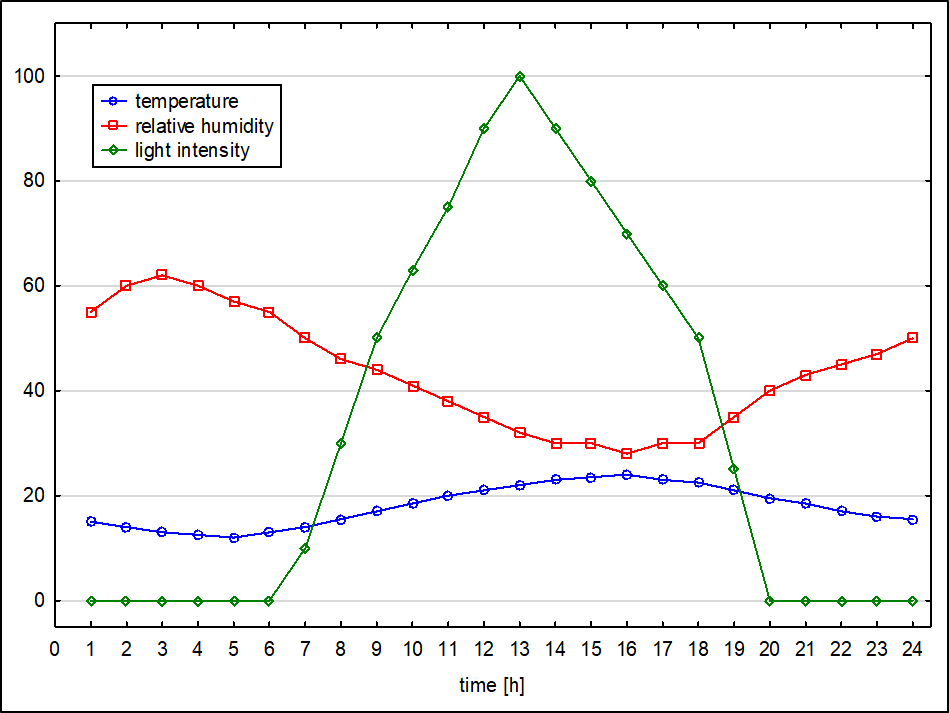


**Figure S1:** Daily course of temperature [°C], relative humidity [%], and light intensity [lx] in all hypobaric chambers, irrespective of applied air pressure.

**Figure S2:** Numbers of prokaryote (**A**) and fungal (**B**) observed species in the rhizosphere of *B. rupestre*, *H. pilosella*, and *T. pratense* grown at different air pressure levels (98, 85, 75 and 62 kPa). Individual data points are shown along with dashed lines representing separate linear regression fits. The *p-*values obtained from separate general linear models indicate that there is no statistically significant relationship (*p* > 0.05) between the number of observed species and air pressure.

**Figure S3:** Prokaryote (**A**) and fungal (**B**) Shannon Index in the rhizosphere of *B. rupestre*, *H. pilosella*, and *T. pratense* grown at different air pressure levels (98, 85, 75 and 62 kPa). Individual data points are shown along with dashed lines representing separate linear regression fits. The *p*-values obtained from separate general linear models indicate that there is no statistically significant relationship (*p* > 0.05) between the Shannon Index and air pressure.

**Figure S4:** Prokaryote (**A**) and fungal (**B**) community structure of rhizosphere samples incubated at the air pressure level of the original sampling location (85 kPa, control approach) from represented by NMDS-plots based on Bray-Cutis dissimilarities for different plant species (*B. rupestre*, *H. pilosella*, *T. pratense*). Plant species are indicated by different colors and *p* values refer to differences between plant species (PERMANOVA analysis). The stress value (shown in the top right corner of the plots) indicates the goodness of fit of the ordinations.

**Figure S5:** Barplots showing relative abundances of the 10 most abundant prokaryote (**A**) and fungal (**B**) orders in the rhizosphere of three plant species (*B. rupestre*, *H. pilosella*, *T. pratense*).

**Figure S6:** Prokaryote (**A**) and fungal (**B**) biomarker indicative for different plant species (*B. rupestre*, *H. pilosella*, and *T. pratense*) were identified with ALDEx2 based on a Kruskal-Wallis test, on genus level. The left panel shows biomarker genera for different plant species (indicated by different colours) and respective *p*-values (P.adjust = posterior Benjamini-Hochberg corrected *p-*values). The relative abundances of these genera are represented by a heatmap (right panel).
